# Radiotherapy-Related Gene Signature in Prostate Cancer

**DOI:** 10.3390/cancers14205032

**Published:** 2022-10-14

**Authors:** Sotirios P. Fortis, Maria Goulielmaki, Nicolas Aubert, Panagiota Batsaki, Sotirios Ouzounis, Dionisis Cavouras, Gilles Marodon, Savvas Stokidis, Angelos D. Gritzapis, Constantin N. Baxevanis

**Affiliations:** 1 Cancer Immunology and Immunotherapy Center, Cancer Research Center, Saint Savas Cancer Hospital, 11522 Athens, Greece; 2 Centre d’Immunologie et Maladies Infectieuses-Paris, CIMI-PARIS, Sorbonne Université, INSERM, CNRS, 75013 Paris, France; 3 Department of Biomedical Engineering, University of West Attica, 12243 Athens, Greece; 4 Institute of Chemical Biology, National Hellenic Research Foundation, 11635 Athens, Greece

**Keywords:** prostate cancer, radiotherapy, immune system, gene-expression, six-gene signature, predictive biomarker

## Abstract

**Simple Summary:**

Radiation therapy (RT) is an established therapeutic regimen for prostate cancer patients which aims for the direct elimination of tumor cells in the prostate gland and occasionally at distant anatomic sites. In this study, we performed next-generation sequencing-based gene expression analysis in peripheral blood from prostate cancer patients obtained pre- and post-radiotherapy and found six independently down-regulated genes including *CCR7*, *FCGR2B*, *BTLA*, *CD6*, *CD3D*, and *CD3E*. The analysis of the expression of the 6-genes as a signature also revealed significantly lower levels post- vs. pre-radiotherapy. Data extracted from the PRAD (PRostate ADenocarcinomas) dataset linked low levels of the 6-gene signature to better survival. More importantly, this 6-gene signature strongly correlated with a favorable prognosis regardless of poor standard clinicopathological parameters (i.e., Gleason score ≥ 8 and T3), thus highlighting its potential predictive value.

**Abstract:**

Radiotherapy for localized prostate cancer has increased the cure and survival rates of patients. Besides its local tumoricidal effects, ionizing radiation has been linked to mechanisms leading to systemic immune activation, a phenomenon called the abscopal effect. In this study, we performed gene expression analysis on peripheral blood from prostate cancer patients obtained post- radiotherapy and showed that 6 genes, including *CCR7*, *FCGR2B*, *BTLA*, *CD6*, *CD3D*, and *CD3E*, were down-regulated by a range of 1.5–2.5-fold as compared to pre-radiotherapy samples. The expression of the signature consisting of these six genes was also significantly lower post- vs. pre-radiotherapy. These genes are involved in various tumor-promoting immune pathways and their down-regulation post-radiotherapy could be considered beneficial for patients. This is supported by the fact that low mRNA expression levels for the 6-gene signature in the prostate tumor tissue was linked to better survival. Importantly, we report that this 6-gene signature strongly correlated with a favorable prognosis regardless of poor standard clinicopathological parameters (i.e., Gleason score ≥ 8 and T3 (including T3a and T3b). Our pioneering data open the possibility that the 6-gene signature identified herein may have a predictive value, but this requires further long-term studies.

## 1. Introduction

Targeted therapeutics constitute a major part among multifaceted cancer treatments [1]. Such targeted therapies among others may also involve immune checkpoint or kinase inhibitors which act to activate antitumor immune reactivity either directly by reinvigorating the exhausted endogenous antitumor immunity or indirectly by neutralizing over-activated oncogenic pathways [2,3]. These agents may have a greater impact on clinical outcomes if combined with treatment modalities that down-regulate the expression of immune suppressor genes.

Although prostate cancer (PCa) has survival rates of more than 90% after 5 years, it is still one of the major causes of cancer-related death among men in western countries due to its high incidence [4]. Radiation therapy (RT) is an established treatment option for the management of localized PCa (LPCa) [5], which aims to directly kill tumor cells in the prostate gland and occasionally at distant anatomic sites [6]. There is accumulating evidence to suggest that RT generates local and also systemic immune-based pathways variously [7,8], but mostly via the release of tumor antigens which are specifically recognized by T cells and induce downstream signaling [9,10,11]. However, so far, there is no report on the identification of genes that are affected by RT and are involved in immune activation mechanisms controlling tumor progression. Therefore, the present study aimed to investigate the alterations in immune response genes in the peripheral blood of PCa patients with localized disease post-RT, and to analyze the correlation of these changes with the incidence of recurrences from the Prostate Adenocarcinoma (PRAD) dataset. Thus, the potential mechanisms of immune activation induced by RT might be clarified, which may improve our understanding of tumor immune surveillance.

## 2. Materials and Methods

### 2.1. Selection of Patients and Samples

Twenty-three PCa patients were enrolled between 1/2019–6/2020. The clinicopathological characteristics are summarized in Table 1. All the patients had acinar adenocarcinoma of the prostate. Seventeen patients received primary RT, while the remainders (n = 6) received RT post-radical prostatectomy (adjuvant RT). Only the patients who completed the radiation schedule without breaks or dose reductions were eligible for the study. All six patients who were treated with adjuvant RT after radical prostatectomy had positive margins. Second, all patients who were treated with primary RT (n = 17) or adjuvant RT after radical prostatectomy (n = 6) were already under androgen-deprivation therapy (ADT) during the past six months (and therefore at the first blood sampling (i.e., pre-RT)).

Blood was collected before the initiation of RT and 90 days after the end of RT. The blood sampling was performed by the medical doctor and the blood was collected in a tube containing K2E (EDTA). This time point (i.e., 3 months) was chosen based on previous findings, reporting that by this time the immune cell populations have recovered to their normal numbers before the initiation of therapy [12,13]. Patient follow-up ranged between 22 and 39 months depending on the patient’s enrolment date.

### 2.2. Ethics Approval

The study was conducted in accordance with the Declaration of Helsinki, and all of the participants provided written informed consent. The study was approved by the Saint Savas Cancer Hospital Institutional Review Board (approval no. IRB-ID6777/14-06-2017) and the Ethical Committee of the Medical School of the National and Kapodistrian University of Athens (approval no. ID247/28-01-2020).

### 2.3. Isolation of RNA

RNA was extracted from peripheral blood using the PureLink™ Total RNA Blood Kit (Invitrogen, Thermofisher, Waltham, MA, USA) within thirty minutes after the blood collection. As a next step, we performed DNase treatment using ezDNase™ Enzyme (Invitrogen, Thermofisher, Waltham, MA, USA) to ensure that there were no contaminations with DNA. The quantification of the extracted RNA was performed by a Qubit Fluorometer 3.0 (Thermofisher, Waltham, MA, USA), which detects fluorescent dyes specific to the RNA. The quantity of the input was 10 ng of total RNA, which was used for manual library preparation. The extracted RNA was stored at −80 °C.

### 2.4. Oncomine Immune Response Research Assay

The RNA-based Next Generation Sequencing (NGS) panel Oncomine Immune Response Assay (OIRRA) (Thermofisher, Waltham, MA, USA) was adopted to measure the expression levels of immune-related genes. This panel allowed the simultaneous evaluation of 398 genes related to immune system activation, including genes associated with adhesion, migration, TCR co-expression, checkpoint pathways, cytokine signaling, dendritic cells, macrophage, lymphocyte infiltrate, and B cell markers. The RNA-sequencing analysis was performed on RNA from the peripheral blood of the patients. In detail, cDNA synthesis was performed using SuperScript™ VILO™ cDNA Synthesis Kit (Invitrogen, Thermofisher, Waltham, MA, USA) for the preparation of cDNA target amplification reactions. For library preparation, Ion AmpliSeq™ Library Kit 2.0 (Ion Torrent™, Thermofisher, Waltham, MA, USA) and Ion Xpress™ Barcode Adapters (Ion Torrent™, Thermofisher, Waltham, MA, USA) were used and AMPure XP (Beckman Coulter, Brea, CA, USA) beads were employed for purification and size selection throughout the workflow. Subsequently, qPCR was performed for library quantification, using an Ion Library TaqMan™ Quantitation Kit (Ion Torrent™, Thermofisher, Waltham, MA, USA) and a Quantstudio 5.0 Real-Time PCR instrument and software (Thermofisher, Waltham, MA, USA). The concentration per library was 50 pM for Ion Chef Preparation and after dilution and calculation of the proper concentration, the libraries were combined in order to proceed with templating and sequencing. Template preparation and chip loading took place on the Ion Chef System (Thermofisher, Waltham, MA, USA) and the sequencing step was executed using the Ion S5 System (Thermofisher, Waltham, MA, USA). The targeted RNA-sequencing analysis was obtained using the Ion Torrent Immune Response RNA plugin that produced gene transcript data.

### 2.5. Gene Expression Analysis and Statistics

The Read Per Million data (RPM) were log-transformed and normalized and the gene-level count data generated from the run were further analyzed with the Affymetrix Transcriptome Analysis Console (TAC) software. Results normalized by RPM were downloaded from the Immune Response RNA plugin and then uploaded to the TAC software. The Read cutoff per sample was 1.5 million total reads. Gene expression analysis and graph preparation were performed in GraphPad Prism 8.0.2 for Windows (GraphPad Software, Inc., San Diego, CA, USA), using the normalized average log2 values corresponding to gene expression levels, as well as the resulting fold change values. A non-parametric Wilcoxon’s (paired) test was performed for the identification of differences in immune gene expression among patients pre- and post-RT. Data are presented as the median with range. A correlation matrix analysis (Pearson coefficient) was used to compute the correlation between the six genes that constitute the proposed signature, before and post-RT. P values below 0.05 were considered to indicate a statistically significant difference. The interaction network of the 6-GS was built using the GeneMANIA database [14]. Accordingly, the interaction network of the respective proteins was built using the STRING database [15]. Gene Ontology was conducted using the GOnet tool, available from [16]. Univariate and multivariate survival analyses (Cox regression) were conducted using IBM SPSS 24 (SPSS Inc., Chicago, IL, USA). For the multivariate analysis, the forward stepwise method with a threshold of 0.05 as an entry point was used. All categorical covariates were transformed into numeric codes as follows: GS: ≤6, 1; 7, 2; ≥8, 3; T status: T1-T2, 1; T3, 2; 6-gene signature: Low expression, 1; High expression, 2.

### 2.6. TCGA PRAD Analysis

The PRAD data from The Cancer Genome Atlas (TCGA) database were extracted using the Xena browser (http://xena.ucsc.edu, accessed on 15 July 2022) provided by the University of California (Santa Cruz, CA, USA). mRNA expression of genes of interest, progression-free interval (PFI), PFI-time and phenotypic data were used (sample_type, pathologic_T-stage, Gleason_score and PSA value). Only primary tumor samples (n = 497) were selected for subsequent analysis. A patient was considered with a high gene expression if the expression level of this was higher than its respective median among all PRAD patients. Otherwise, a patient was considered with a low expression. Then, additional genes of interest were sequentially added to the primary analysis leading to the 6-GS.

## 3. Results

### 3.1. Sample Demographics

Twenty-three patients with LPCa undergoing external beam radiation therapy (EBRT) were enrolled in the study (Table 1). The mean age of the subjects was 73 [range, 53–81] years. Almost half of the patients (n = 12/23; 52.2%) had stage T2 (a–c) disease with Gleason scores ranging from 6 to 9 (mean: 7) and baseline PSA mean levels 18.74 ng/mL (range, 5.51–100.00 ng/mL) that were consistent with intermediate- to high-risk progression of the disease (Table 1) [17]. Seventeen of the participants were receiving primary RT and the remaining six patients had adjuvant RT post radical prostatectomy. The EBRT characteristics are presented in Table 1. Notwithstanding the two groups receiving different treatments, we should note that biochemical control rates in patients with localized PCa treated either with ERBT or with RP appear similar over extended periods of time [18,19]. This would suggest that the tumor burden in these two groups is either similar or, if with minor differences, these should not interfere with molecular or phenotypical alterations in peripheral blood. Therefore, we analyzed the data from these 2 groups jointly.

### 3.2. Gene Expression and Survival

**Figure 1:**
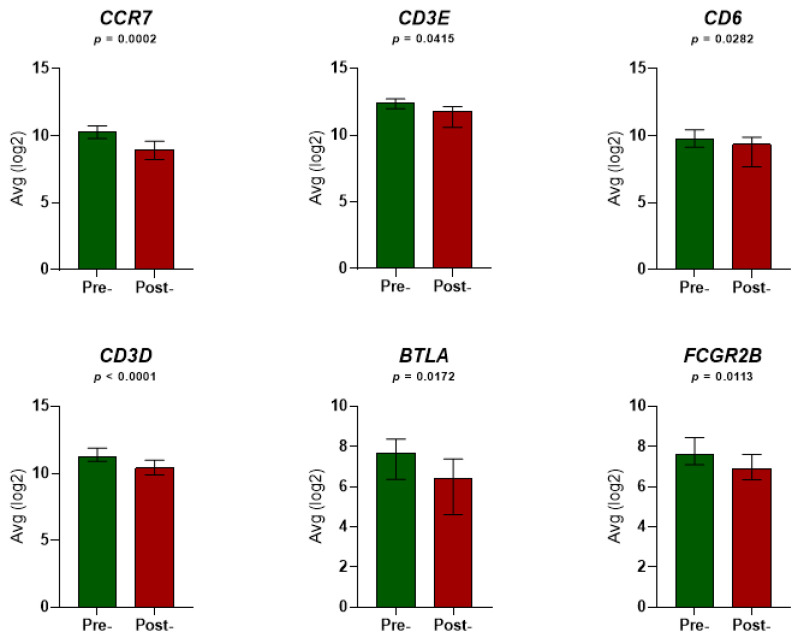
Expression levels of the six immune-related genes in the peripheral blood of 23 localized prostate cancer (LPCa) patients before and after radiation therapy (RT). Each graph corresponds to expression level alterations of each single gene pre-RT and post-RT. Each column illustrates the average mRNA levels of each gene normalized by RPM and log2-transformed. Non-parametric Wilcoxon’s (paired) test was performed to indicate whether the expression levels pre-RT and post-RT differed significantly among patients. The error bars denote the median values with interquartile range. P values below 0.05 indicate statistical significance. Avg: average.

Six genes related to (a) immune checkpoints (n = 2; *BTLA* and *CD6*); (b) regulatory macrophages and T cells (n = 2; *CCR7* and *CD3D*); and (c) immune deficiencies and unfavorable prognosis (n = 2; *CD3E* and *FCGR2B*) were down-regulated post-RT compared with pre-RT (statistical *p*-range: <0.0001–0.0415; Figure 1).

**Figure 2:**
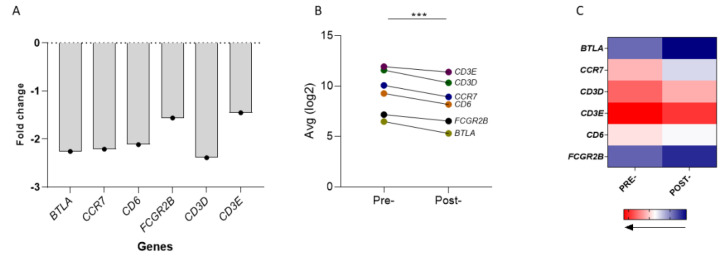
Alterations in gene expression of the 6-GS in the peripheral blood of 23 LPCa patients before and after RT. (**A**) Scatter dot plot representing fold change (FC) in the six genes pre-RT and post-RT. FC values were generated by conversion of the normalized average log2 values corresponding to gene expression levels pre-RT and post-RT. Data are presented as means. (**B**) The 6-GS is significantly downregulated post-RT (*** *p* < 0.0001). The depicted lines represent the conversion of each described gene from higher (pre-RT) to lower levels (post-RT). (**C**) Heat map showing the range of differential expression levels of the six genes. For the analysis, the median expression levels of each gene, pre-RT and post-RT, were used. The expression level values were normalized by RPM, log2-transformed and then median-centered for each patient. Different colors indicate different levels of gene expression: from red (high expression, max. value 12.43) to blue (low expression, min. value 6.42).

Four of the six genes (*BTLA*, *CCR7*, *CD6* and *CD3D*) were between 2.0-fold and 2.5-fold down-regulated, whereas *FCGR2B* and *CD3E* were down-regulated by 1.56-fold and 1.45-fold, respectively (Figure 2A). Moreover, the 6-genes jointly analyzed as a signature, were expressed at significantly lower levels post-RT vs. pre-RT (*p* < 0.0001; Figure 2B). The heat map demonstrated the differential gene expression levels in the two time-periods (Figure 2C).

Due to the extended time-period required for survival analyses in patients with LPCa [20], we explored the clinical significance of our data using the PRAD dataset. In this database, information from 498 primary tumor samples is available for analysis. For one patient there were no follow-up data. Of the remaining 497 patients, only three patients (<1%) had confirmed metastatic disease. Thus, the PRAD-cohort including almost entirely patients with localized disease was comparable to our group.

**Figure 3:**
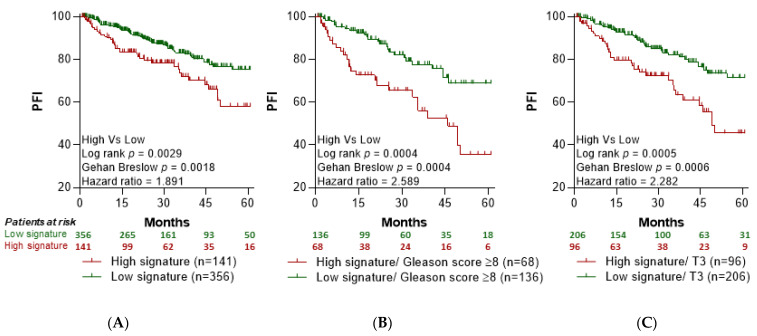
The 6-GS is associated with lower progression-free intervals in PCa patients. Kaplan-Meier curves for Progression Free Interval (PFI) in PCa patients were designed based on the dichotomized median expression of the 6-GS in prostate tumor tissue. (**A**) Association between the 6-GS expression levels and PFI in PCa patients. (**B**) Association between the 6-GS expression levels and PFI among PCa patients with Gleason score ≥8. (**C**) Association between the 6-GS expression levels and PFI among PCa patients with T3 (3a,3b) stage tumors. The data were extracted from the PRAD (PRostate ADenocarcinomas) dataset. The red lines indicate cases with high expression (expression value > median), while the green lines indicate cases with low expression (expression value < median) of the 6-GS. Moreover, the number of patients at risk are presented in the graph.

We compared PFI in LPCa patient groups stratified by the 6-GS levels in prostate tumor tissue above vs. below median (simulating the higher vs. decreased expression levels of this signature in patients’ peripheral blood at pre-RT and post-RT, respectively). As shown in Figure 3A, PFI was significantly higher in the group of patients with levels of the 6-GS below median vs. those expressing 6-GS levels above median. We next examined survival in PCa patients with poor prognosis based on standard clinicopathological parameters including patients with Gleason score ≥ 8 and T-stage T3. Also, in this case, PFI was markedly higher in patients with the 6-GS expression levels below median than above median (Figure 3B,C).

**Figure 4:**
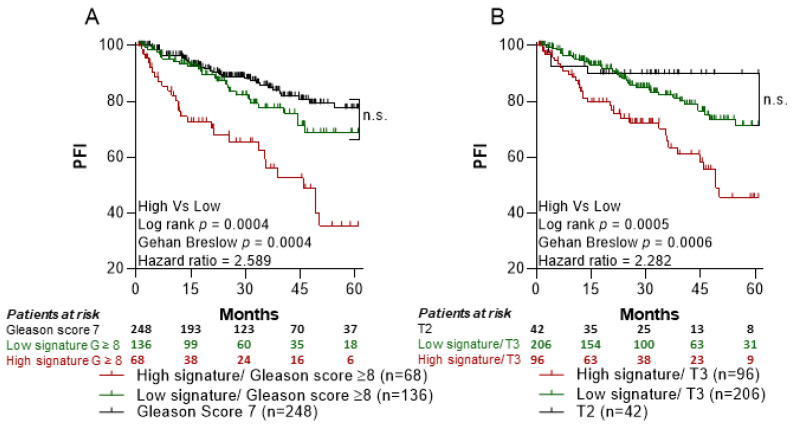
Prognostic potential of the 6-GS in PCa patients. Kaplan-Meier curves for Progression Free Interval (PFI) were designed based on the median expression of the 6-GS in prostate tumor tissue in association with poor clinicopathological parameters. Moreover, the number of patients at risk are presented in the graph. (**A**) No significant difference in PFI among patients with high Gleason score (≥8) and low expression (below median) of the 6-GS (green line; n = 136) and patients with intermediate Gleason score (7) (black line; n = 248). In the same lines, (**B**) the PFI in the group of patients with unfavorable T3 stage (3a,3b) and low expression of the 6-GS (green line, n = 206) did not differ statistically as compared to PFI in patients with more favorable T2 stage (2a–2c) (black line, n = 42). Patients with high expression of the 6-GS (above median) having either Gleason score ≥8 (**A**) or T3 stage (3a,3b) (**B**) (red lines; n = 68 and n = 96 for A and B, respectively) had a significantly worse PFI as compared to patients with low expression of the 6-GS (green lines). n.s.: non significance.

Importantly, PFI for patients with high risk of recurrence based on the standard clinicopathological parameters (i.e., T3 or Gleason score ≥8) but having the 6-GS levels below median, did not statistically differ from PFI observed in patients with more favorable clinicopathological parameters (i.e., Gleason score = 7 or T2; Figure 4A,B, respectively).

To investigate the prognostic significance of the 6-GS, we conducted univariate and multivariate analyses in the results extracted from the PRAD database, using established clinicopathological factors (i.e., Gleason score and T stage) and our 6-GS as covariates and 5-years PFI as endpoint. In the univariate analysis (Table 2), a statistically strong impact for the patients expressing high levels of the 6-GS (*p* = 0.003) on PFI was revealed. In addition, the statistical significance of the 6-GS was stronger than the T stage (*p* = 0.005). In the multivariate analysis, the prognostic significance of the three covariates (T stage, Gleason score and 6-GS) was analyzed (Table 2). After stepwise selection, the 6-GS was found to be a strong prognosticator for the PFI (*p* = 0.006). However, as expected, the Gleason score remained a stronger prognostic factor (*p* < 0.001).

**Figure 5:**
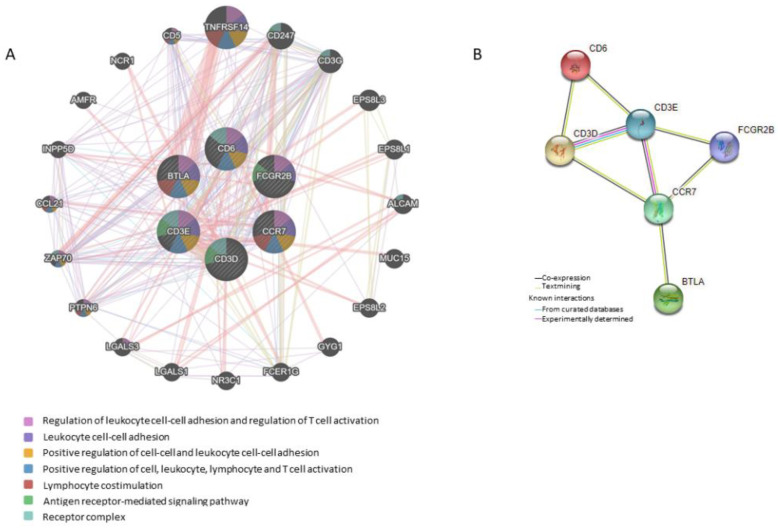
Functional network analysis of the six differentially expressed genes post-RT in LPCa patients. (**A**) Gene interaction network built on the 6-GS using the GeneMANIA database. The different color of the lines connecting the relevant genes depicts the type of interaction, including (1) regulation of leukocyte cell-cell adhesion and regulation of T-cell activation; (2) leukocyte cell-cell adhesion; (3) positive regulation of cell-cell adhesion and positive regulation of leukocyte cell-cell adhesion; (4) positive regulation of cell, leukocyte, lymphocyte and T-cell activation; (5) lymphocyte co-stimulation; (6) antigen receptor-mediated signaling pathway; and (7) receptor complex function. (**B**) Internal interactions between the 6-GS expression products. The corresponding proteins were mapped in the protein-protein interaction (PPI) network using the STRING database. Nodes and edges represent proteins and their interactions, respectively. Filled, colored nodes represent query proteins and first shell of interactors with known or predicted 3D structures. Turquoise and pink edges show known interactions extracted from curated databases or those experimentally determined, respectively. Yellow edges represent text-mining interactions, black edges indicate that the respective proteins are co-expressed.

**Figure 6:**
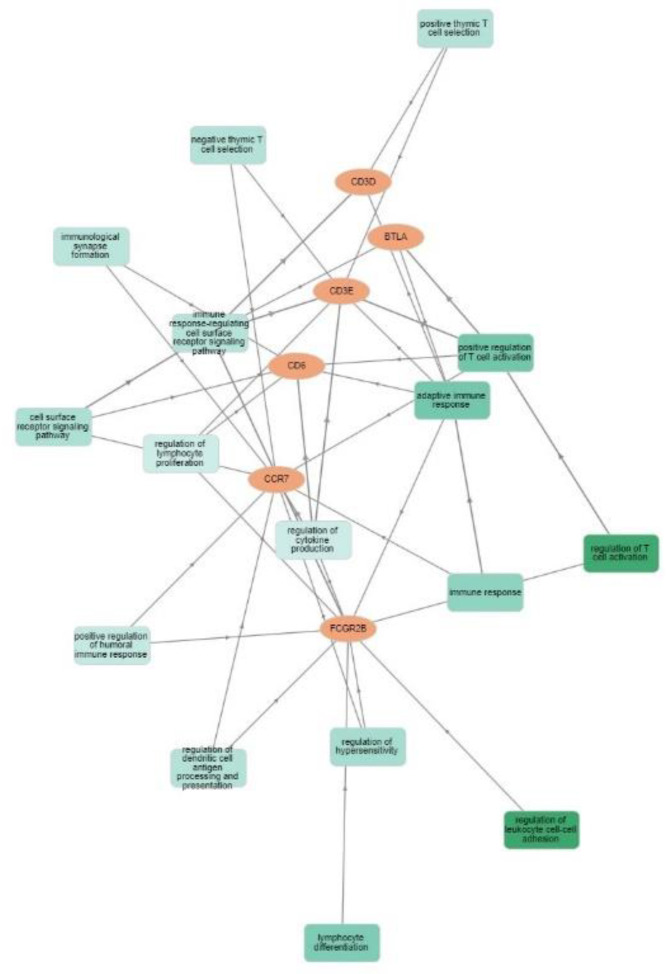
Visualization of Gene Ontology (GO) enrichment profiles of the 6-gene signature using the GOnet tool (only gene-connected terms, *p*-value threshold ≤ 7.22 × 10^−5^). The orange-colored nodes represent the 6 downregulated genes, while the green-colored nodes represent the functional groups (GO term/pathway nodes). The GO term/pathway network connectivity is defined by edges. GO term nodes are colored according to *p*-value of enrichment. The dark green nodes show higher statistical significance than the light green nodes (regulation of leukocyte cell-cell adhesion; *p*-value = 4.10 × 10^−6^ and inflammatory response; *p*-value = 2.88 × 10^−1^, respectively).

Functional networks of the six differentially expressed genes were examined to determine the involved pathways. The network analysis describes pathways that are related to (1) regulation of leukocyte cell-cell adhesion and regulation of T-cell activation; (2) leukocyte cell-cell adhesion; (3) positive regulation of cell-cell adhesion and positive regulation of leukocyte cell-cell adhesion; (4) positive regulation of cell, leukocyte, lymphocyte and T-cell activation; (5) lymphocyte co-stimulation; (6) antigen receptor-mediated signaling pathway; and (7) receptor complex function (Figure 5A). The most important linkage was found between *TNFRSF14 (HVEM)* and the 6-GS (through *BTLA*). We also investigated the internal interactions of RT-downregulated gene products by mapping them to the PPI network using the STRING database [15]. The minimum required interaction score was set at 0.400 (medium confidence). The results showed that CD3E had the strongest correlation with the majority of the other gene products, followed by CD3D and CCR7 (Figure 5B). More specifically, the analysis revealed an average node degree (average number of edges per node) of 2.67, with an average local clustering coefficient (strength of the connection between adjacent nodes, ranging between 0 and 1) of 0.75 and a PPI enrichment *p*-value of 1.23 × 10^−9^ (significance of interactions). These parameters indicate that the resulting network has significantly more interactions than would be expected for a random set of proteins of the same size and degree of distribution. Text-mining interactions and co-expression were revealed between certain proteins (CD6/CD3D, CD3D/CD3E, CD6/CD3E, CD3E/FCGR2B, CD3E/CCR7, CD3D/CCR7, FCGR2B/CCR7, CCR7/BTLA). Figure 6 shows the six downregulated genes and the functional groups (GO term/pathway nodes) they relate to. The respective *p*-values and gene interconnections are shown in Table S1.

## 4. Discussion

Novel platforms are emerging as valuable tools for the diagnosis, longitudinal patient monitoring and disease prognosis, as well as for the prediction of response to treatment. Among these, the stable adoption of liquid biopsy, novel immunohistochemical assays and gene-related signatures seem to be promising across urological malignancies. For example, the qualitative and quantitative characterization of circulating tumor cells (CTCs) identified by liquid biopsy has been proven more reliable than PSA in predicting the survival of metastatic prostate patients [21] while the size of the tumor fraction in cell-free DNA is related to disease staging and tumor burden [22]. In patients with bladder cancer, quantification of CTCs before radical cystectomy can be a strong prognosticator of disease recurrence and overall survival [23]. Moreover, as recently reviewed by Casanova-Salas et al., CTCs genetic profiling holds strong predictive potential for the identification of responders among prostate patients that receive androgen receptor signaling inhibitors (ARSI), PARP inhibitors and other therapeutic regimes [22]. Similarly, Yazgan et al. found a pan-immune-inflammation value derived from total blood cell counts before treatment with ARSI with marked prognostic potential in patients with metastatic castration-resistant prostate cancer [24]. Regarding immunotherapy, PD-L1 is becoming quite popular as both genetic [25] and immunohistochemical [26] marker with prognostic and/or predictive roles in renal cancer patients treated with immune checkpoint inhibitors. Finally, DNA damage response parameters are widely adopted for the prediction of response to certain treatment modalities in patients with advanced prostate cancer [27,28] or urothelial carcinoma [29]. However, there is a gap in knowledge regarding radiation-induced immune alterations which could serve as putative prognostic and/or predictive biomarkers in PCa.

RT, in addition to its direct tumoricidal effects, additionally exerts indirect antitumor effects via systemic immune activation resulting in an active immunosurveillance against non-irradiated tumor cells. The immune-activating effects of the ionizing radiation are thought to be primarily mediated via the release of tumor antigens from the dying tumor cells which act as an in situ vaccine for tumor peptide-specific T-cell priming [11]. Other immunopotentiating effects of RT include, but are not restricted to, M1 macrophage and T-cell accumulation into the tumor as well as the release of immunostimulatory adjuvants locally, all of which support the combination of RT with immunotherapy [30,31,32,33,34]. Clinical studies have also reported distant responses in patients receiving RT in combination with immune checkpoint inhibitors which were associated with alterations in circulating lymphocyte subsets and antibody responses to tumor-associated antigens [35,36,37,38]. In addition, there are studies to show changes in circulating immune cell subpopulations and cytokines in cancer patients after RT who did not receive systemic treatment [39,40]. However, so far there is no report to show an effect of RT on the expression of genes involved in immune regulation pathways affecting tumor progression which could further contribute to its immune-activating effects. Investigating differences in the expression of such genes in circulation post-RT could be critical for the design of novel immunotherapy trials in combination with RT.

The six critical genes, including *CCR7*, *FCGR2B*, *BTLA*, *CD6*, *CD3D* and *CD3E*, play important roles in favoring tumor progression and promoting negative immune-regulatory effects. For example, CCR7 and its ligands (CCL19/CCL21) are a vital axis for carcinogenic properties, such as epithelial-mesenchymal transition, tumor invasion and migration [41,42]. In addition, high numbers of peripheral CD8+ T-cells expressing differentiation markers and lacking *CCR7* are associated with response to nivolumab in NSCLC patients [43] whereas the presence of CD8+CCR7+ T-cells in the peripheral blood has been demonstrated to associate with disease recurrences in patients with head and neck cancers [44]. Moreover, the silencing of CCR7, a protein involved in angiogenesis, inhibits prostate cancer cell proliferation, migration and invasion [45]. *FCGR2B* expressed by myeloid effector cells inhibits direct tumor cell depletion by therapeutic antibodies via competition with its activating FCGR counterparts. In addition, FCGR2B on malignant B-cells has been found to advance the internalization of targeting monoclonal antibodies, counteracting their capacity to generate antibody-dependent cellular cytotoxicity and thus diminishing their therapeutic efficacy [46]. Interestingly, in a melanoma mouse model, *FCGR2B* has been demonstrated to be upregulated in tumor-infiltrating CD8+ T-cells hampering their antitumor efficacy [47]. In the same study, it was also shown that CD8+ T-cells in melanoma patients express *FCGR2B*, proposing its role in down-regulating tumor-directed immune responses in humans. Increased levels of circulating BTLA or its high expression on CD8+ and CD4+ T-cell subsets have been reported to associate with poor prognosis in many types of malignant diseases [48,49,50,51]. More specifically, it has been shown that the reduction of *BTLA* expression in tumor infiltrating lymphocytes (TILs), through targeting *HVEM*, might result in better tumor control in a humanized mouse model of PCa [52]. Moreover, IFNγ production by tumor-specific CD8+ T-cells in response to melanocytic antigens has been shown to be impeded via BTLA signaling [53]. *CD6* functions as an immune checkpoint mainly expressed by lymphocytes including T and natural killer (NK) cells [54,55]. CD6 interacts with its ligands on cancer cells restricting adaptive and innate antitumor immune responses [55]. Blocking this interaction with an anti–human CD6 monoclonal antibody resulted in the enhanced killing of tumor cell lines in vitro by NK and CD8+ T-cells mostly via upregulation of the activating receptor NKG2D and increased expression levels of perforin/Granzyme B in parallel with a reduction of the inhibitory NKG2A receptor [55]. In the same study, it was shown that the blocking of CD6 in vivo resulted in the rapid rejection of breast cancer xenografts.

*CD3D* upregulation has been associated with resistance to anti-tumor therapy in patients with uveal melanoma, which was attributed to tumor infiltration by increased numbers of immune-suppressive regulatory T-cells [56]. The respective resistance mechanism involves the upregulation of indoleamine 2,3-dioxygenase and its interaction with IFNγ, leading to the promotion of tumor immunosuppression through regulatory T-cell-dependent recruitment of myeloid-derived suppressor cells [56,57]. Moreover, the downregulation of *CD3D* in the peripheral blood of PCa patients undergoing EBRT was correlated with CD8+ T-cell suppressed responses leading to the development of cancer-related fatigue during radiation therapy [58]. *CD3E* expression is upregulated in certain cancer types [59] and it has been recently highlighted as an attractive therapeutic target in cancer [60,61]. Preclinical studies in melanoma-bearing syngeneic mice revealed that the concomitant blockade of CD3E and TRP-1 facilitates tumor shrinkage through the enhanced influx of innate and adaptive immune cells [61]. A bioinformatics analysis using TCGA data from bladder cancer patients found *CD3E* to be downregulated in the luminal compared to basal tumor samples, with the luminal subtype being associated with better survival rates. In the same study, *CD3E* was highlighted as a substantial regulator of the tumor microenvironment, since CD4+ memory T-cells and regulatory T-cells were found to be negatively correlated with *CD3E* expression levels [60], while in low-grade glioma patients, *CD3E* upregulation served as a marker of significantly worse prognosis [62]. The possibility that downregulation of *CD3D* and *CD3E* genes post-RT could be attributed to an RT-based decreasing effect on circulating T-cell frequencies is unlikely since the blood sampling was performed at 3 months post-RT, by which time-point the lymphocyte numbers have been recovered to normal levels pre-RT [12,13].

PCa patients with localized disease receiving primary or adjuvant RT have an extended 5-years PFI which, as extrapolated from various studies, ranges between 90–100%, 80–95% and 60–87% for patients in the low-risk, intermediate-risk, and high-risk groups, respectively [18,19,63,64,65,66]. Consequently, it was not possible to do correlations with clinical outcomes because of the too short follow-up time (22–39 months) between the completion of RT and the writing of this manuscript. Therefore, we performed analyses in the PRAD dataset which revealed that below-median expression of the 6-GS mRNA was associated with a longer PFI over 5 years whereas a reduced PFI was seen in patients with above-median expression of the 6-GS. Notably, such correlations were found in groups of patients with poor clinicopathologic characteristics including Gleason score ≥ 8 and T-stage 3. Importantly, PFI for these patients with a high risk of recurrence having the favorable low expression 6-GS were almost indistinguishable from those observed in patients with more favorable clinicopathological parameters (i.e., Gleason score 7 and T-stage 2). We may propose that RT by lowering the expression of the 6-GS reinforces antitumor immunity resulting in slow tumor growth rates, thus increasing PFI. The possibility that the alterations in gene expression could be attributed to the ADT is highly unlikely simply due to the fact that all 23 PCa patients had been receiving ADT for 6 months before RT implying that these gene alterations should have been detected pre-RT. Nonetheless, we cannot formally exclude the possibility that such alterations could be the result of a combined effect among these two therapeutic modalities. This study has limitations due to the low number of patients examined and because it lacked experimental and clinical studies to validate the function of our 6 GS. With regard to the second limitation, the functional programme of these six genes could be extrapolated from the studies cited above, which demonstrate their role as critical components of various molecular pathways participating to the immune control of tumor progression. Regardless, more patients are needed to be examined along with further studies to substantiate the predictive value of this signature.

## 5. Conclusions

In conclusion, by comprehensively analyzing the immune gene expression levels in the peripheral blood of LPCa patients at baseline and post-RT, our study developed a model based on six genes related to immune suppression and tumor-promoting pathways. It is expected that the 6-GS will predict the prognosis of PCa patients not only with localized disease but also of patients at more advanced PCa stages. 

## Figures and Tables

**Table 1:** Clinicopathological characteristics of the patients and details of the radiation therapy.

Patients with Localized PCa (n = 23)	
Characteristics of Initial PCa Staging	
Median Age at Diagnosis (Years) (Range)	73 (53–81)
**PSA**	
Mean	18.74 ng/mL
SD	20.85 ng/mL
Range	5.51–100.00 ng/mL
**Gleason Score**	
Mean	7
SD	1
Range	6–9
**T**	
T1c	3 (13.0%)
T2a, T2b, T2c	12 (52.2%)
T3a, T3b	8 (34.8%)
**Type of radiation therapy**	
Primary	17 (73.9%)
Adjuvant	6 (26.1%)
**External beam radiation therapy (EBRT) characteristics**	
**3D Conformal Radiotherapy**	
Median daily dose; Gy (range)	2 (1.8–2.2)
Median total dose; Gy (range)	70 (66–72)
Median Radiation treatment schedule; days (range)	37 (35–38)

**Table 2:** Univariate and multivariate survival analyses (Cox regression) were conducted by IBM SPSS 24. For the multivariate analysis, the forward stepwise method with a threshold of 0.05 as an entry point was used.

Univariate	PFI		
	*p*	Hazard Ratio	HR (95.0% CI)
**T status**	0.005	2.032	1.233–3.349
**Gleason Score**	<0.001	2.389	1.621
**6-gene Signature**	0.003	1.891	1.234–2.898
Multivariate	PFI		
	*p*	Hazard Ratio	HR (95.0% CI)
Model Before Stepwise Selection			
**T status**	0.312	1.324	0.768–2.281
**Gleason Score**	<0.001	2.137	1.405–3.250
**6-gene Signature**	0.007	1.808	1.179–2.772
Model after Stepwise Selection			
**Gleason Score**	<0.001	2.337	1.588–3.439
**6-gene Signature**	0.006	1.828	1.193–2.802

## Data Availability

The data presented in this study are available upon reasonable request.

## Supplementary Materials

The following supporting information can be downloaded at: https://www.mdpi.com/article/10.3390/cancers14205032/s1, Table S1: Gene Ontology (GO) enrichment profiles, including the respective *p*-values and gene interconnections, of the 6-gene signature using the GOnet tool.
