# Correction: Kogionou et al. Radiotherapy-Related Gene Signature in Prostate Cancer. *Cancers* 2022, *14*, 5032

**DOI:** 10.3390/cancers15143616

**Published:** 2023-07-14

**Authors:** Paraskevi Kogionou, Sotirios P. Fortis, Maria Goulielmaki, Nicolas Aubert, Panagiota Batsaki, Sotirios Ouzounis, Dionisis Cavouras, Gilles Marodon, Savvas Stokidis, Angelos D. Gritzapis, Constantin N. Baxevanis

**Affiliations:** 1 Cancer Immunology and Immunotherapy Center, Cancer Research Center, Saint Savas Cancer Hospital, 11522 Athens, Greece; pkogionou@gmail.com (P.K.); fortis@ciic.gr (S.P.F.); mgoulielmaki@eie.gr (M.G.); pmpatsaki@agsavvas-hosp.gr (P.B.); savstok@gmail.com (S.S.); agkritzapis@agsavvas-hosp.gr (A.D.G.); 2 Center for Basic Research, Biomedical Research Foundation of Academy of Athens (BRFAA), 11527 Athens, Greece; 3 Centre d’Immunologie et Maladies Infectieuses-Paris, CIMI-PARIS, Sorbonne Université, INSERM, CNRS, 75013 Paris, France; nicolas.aubert@etu.upmc.fr (N.A.); gilles.marodon@inserm.fr (G.M.); 4 Department of Biomedical Engineering, University of West Attica, 12243 Athens, Greece; souzounis@uniwa.gr (S.O.); cavouras@uniwa.gr (D.C.); 5 Institute of Chemical Biology, National Hellenic Research Foundation, 11635 Athens, Greece

The authors wish to make the following corrections to this paper [1]:

## Addition of an Author

Ms. Paraskevi Kogionou (P.K.) was not included as a first co-author in the original publication. The corrected Author Contributions Statement appears here.

Conceptualization, C.N.B.; Data curation, P.K. S.P.F., M.G., P.B. and S.S.; Formal analysis, P.K., S.P.F., M.G., N.A., P.B. and S.O.; Funding acquisition, C.N.B.; Investigation, P.K., S.P.F., M.G., P.B., D.C., G.M., S.S. and A.D.G.; Methodology, P.K., S.P.F., M.G. and S.S.; Project administration, C.N.B.; Resources, S.S., D.C., G.M. and C.N.B.; Supervision, C.N.B.; Validation, P.K., S.P.F., M.G., G.M. and C.N.B.; Visualization, P.K., S.P.F., M.G., N.A. and S.O.; Writing—original draft, P.K., S.P.F., M.G. and C.N.B.; Writing—review and editing, S.P.F., M.G., N.A., G.M., S.S., A.D.G. and C.N.B. All authors have read and agreed to the published version of the manuscript.

The authors apologize for any inconvenience caused and state that the scientific conclusions are unaffected. This correction was approved by the Academic Editor. The original publication has also been updated.
