# Supplementary material for: Radiotherapy-Related Gene Signature in Prostate Cancer
Source: Cancers (Basel). 2022 Oct 14;14(20):5032. doi: 10.3390/cancers14205032 (PMC9599894; doi:10.3390/cancers14205032)
Supplement: Supplementary file 1 [file cancers-14-05032-s001.zip › cancers-1944878-supplementary.pdf]

.	GO_term_ID	GO_term_def	P	P_FDR_adj	NofGenes	Genes
1	GO:1903037	regulation of leukocyte cell-cell adhesion	4,10E-06	3,04E-02	5	BTLA|CCR7|CD3E|CD6|FCGR2B
2	GO:0050863	regulation of T cell activation	5,50E-06	3,04E-02	5	BTLA|CCR7|CD3E|CD6|FCGR2B
3	GO:0046649	lymphocyte activation	1,49E-05	4,09E-02	5	CCR7|CD3D|CD3E|CD6|FCGR2B
4	GO:0045061	thymic T cell selection	1,50E-05	4,09E-02	3	CCR7|CD3D|CD3E
5	GO:0022407	regulation of cell-cell adhesion	1,85E-05	4,09E-02	5	BTLA|CCR7|CD3E|CD6|FCGR2B
6	GO:0002684	positive regulation of immune system process	3,59E-05	6,59E-02	6	BTLA|CCR7|CD3D|CD3E|CD6|FCGR2B
7	GO:0051249	regulation of lymphocyte activation	6,20E-05	9,75E-02	5	BTLA|CCR7|CD3E|CD6|FCGR2B
8	GO:0002694	regulation of leukocyte activation	1,37E-04	1,56E-01	5	BTLA|CCR7|CD3E|CD6|FCGR2B
9	GO:0045058	T cell selection	1,41E-04	1,56E-01	3	CCR7|CD3D|CD3E
10	GO:0050870	positive regulation of T cell activation	1,50E-04	1,56E-01	4	BTLA|CCR7|CD3E|CD6
11	GO:0002250	adaptive immune response	1,56E-04	1,56E-01	5	BTLA|CD3D|CD3E|CD6|FCGR2B
12	GO:0050865	regulation of cell activation	1,87E-04	1,67E-01	5	BTLA|CCR7|CD3E|CD6|FCGR2B
13	GO:1903039	positive regulation of leukocyte cell-cell adhesion	1,97E-04	1,67E-01	4	BTLA|CCR7|CD3E|CD6
14	GO:0033077	T cell differentiation in thymus	2,50E-04	1,94E-01	3	CCR7|CD3D|CD3E
15	GO:0030155	regulation of cell adhesion	2,64E-04	1,94E-01	5	BTLA|CCR7|CD3E|CD6|FCGR2B
16	GO:0030098	lymphocyte differentiation	2,81E-04	1,94E-01	4	CCR7|CD3D|CD3E|FCGR2B
17	GO:0002682	regulation of immune system process	3,31E-04	2,15E-01	6	BTLA|CCR7|CD3D|CD3E|CD6|FCGR2B
18	GO:0022409	positive regulation of cell-cell adhesion	3,73E-04	2,28E-01	4	BTLA|CCR7|CD3E|CD6
19	GO:0006955	immune response	6,66E-04	3,86E-01	6	BTLA|CCR7|CD3D|CD3E|CD6|FCGR2B
20	GO:0002521	leukocyte differentiation	1,09E-03	5,98E-01	4	CCR7|CD3D|CD3E|FCGR2B
21	GO:0045321	leukocyte activation	1,19E-03	6,22E-01	5	CCR7|CD3D|CD3E|CD6|FCGR2B
22	GO:0051251	positive regulation of lymphocyte activation	1,49E-03	7,46E-01	4	BTLA|CCR7|CD3E|CD6
23	GO:0002883	regulation of hypersensitivity	2,15E-03	1,03E+00	2	CCR7|FCGR2B
24	GO:0002696	positive regulation of leukocyte activation	2,35E-03	1,04E+00	4	BTLA|CCR7|CD3E|CD6
25	GO:0045785	positive regulation of cell adhesion	2,35E-03	1,04E+00	4	BTLA|CCR7|CD3E|CD6
26	GO:0001775	cell activation	2,49E-03	1,06E+00	5	CCR7|CD3D|CD3E|CD6|FCGR2B
27	GO:0050867	positive regulation of cell activation	2,64E-03	1,08E+00	4	BTLA|CCR7|CD3E|CD6
28	GO:0007166	cell surface receptor signaling pathway	3,05E-03	1,20E+00	6	BTLA|CCR7|CD3D|CD3E|CD6|FCGR2B
29	GO:0050776	regulation of immune response	3,58E-03	1,36E+00	5	BTLA|CCR7|CD3D|CD3E|FCGR2B
30	GO:0002604	regulation of dendritic cell antigen processing and presentation	4,22E-03	1,45E+00	2	CCR7|FCGR2B
31	GO:0045059	positive thymic T cell selection	4,22E-03	1,45E+00	2	CD3D|CD3E
32	GO:0045060	negative thymic T cell selection	4,22E-03	1,45E+00	2	CCR7|CD3E
33	GO:0002768	immune response-regulating cell surface receptor signaling pathway	4,91E-03	1,64E+00	4	BTLA|CD3D|CD3E|FCGR2B
34	GO:0043383	negative T cell selection	5,06E-03	1,64E+00	2	CCR7|CD3E
35	GO:0002864	regulation of acute inflammatory response to antigenic stimulus	5,98E-03	1,79E+00	2	CCR7|FCGR2B
36	GO:0001771	immunological synapse formation	5,98E-03	1,79E+00	2	CCR7|CD6
37	GO:0030217	T cell differentiation	6,00E-03	1,79E+00	3	CCR7|CD3D|CD3E

38	GO:0002376	immune system process	7,49E-03	2,17E+00	6	BTLA|CCR7|CD3D|CD3E|CD6|FCGR2B
39	GO:0030097	hemopoiesis	8,43E-03	2,38E+00	4	CCR7|CD3D|CD3E|FCGR2B
40	GO:0002922	positive regulation of humoral immune response	1,04E-02	2,87E+00	2	CCR7|FCGR2B
41	GO:0002764	immune response-regulating signaling pathway	1,13E-02	3,04E+00	4	BTLA|CD3D|CD3E|FCGR2B
42	GO:0048534	hematopoietic or lymphoid organ development	1,19E-02	3,13E+00	4	CCR7|CD3D|CD3E|FCGR2B
43	GO:0002577	regulation of antigen processing and presentation	1,45E-02	3,73E+00	2	CCR7|FCGR2B
44	GO:0002520	immune system development	1,53E-02	3,83E+00	4	CCR7|CD3D|CD3E|FCGR2B
45	GO:0001817	regulation of cytokine production	2,04E-02	4,99E+00	4	CCR7|CD3E|CD6|FCGR2B
46	GO:0002861	regulation of inflammatory response to antigenic stimulus	2,11E-02	5,06E+00	2	CCR7|FCGR2B
47	GO:0050670	regulation of lymphocyte proliferation	2,21E-02	5,15E+00	3	CD3E|CD6|FCGR2B
48	GO:0032944	regulation of mononuclear cell proliferation	2,24E-02	5,15E+00	3	CD3E|CD6|FCGR2B
49	GO:0043368	positive T cell selection	2,49E-02	5,59E+00	2	CD3D|CD3E
50	GO:0070663	regulation of leukocyte proliferation	2,62E-02	5,76E+00	3	CD3E|CD6|FCGR2B
51	GO:0042110	T cell activation	3,07E-02	6,63E+00	3	CCR7|CD3D|CD3E
52	GO:0050778	positive regulation of immune response	4,66E-02	9,87E+00	4	CCR7|CD3D|CD3E|FCGR2B
53	GO:0002673	regulation of acute inflammatory response	7,22E-02	1,50E+01	2	CCR7|FCGR2B
54	GO:0002237	response to molecule of bacterial origin	8,33E-02	1,70E+01	3	CCR7|CD6|FCGR2B
55	GO:0050727	regulation of inflammatory response	9,20E-02	1,84E+01	3	CCR7|CD6|FCGR2B
56	GO:0001954	positive regulation of cell-matrix adhesion	9,72E-02	1,91E+01	2	CCR7|CD3E
57	GO:0048583	regulation of response to stimulus	1,01E-01	1,95E+01	6	BTLA|CCR7|CD3D|CD3E|CD6|FCGR2B
58	GO:0031295	T cell costimulation	1,09E-01	2,07E+01	2	BTLA|CD3E
59	GO:0031294	lymphocyte costimulation	1,13E-01	2,11E+01	2	BTLA|CD3E
60	GO:0050854	regulation of antigen receptor-mediated signaling pathway	1,44E-01	2,61E+01	2	CCR7|FCGR2B
61	GO:0048584	positive regulation of response to stimulus	1,44E-01	2,61E+01	5	CCR7|CD3D|CD3E|CD6|FCGR2B
62	GO:0009988	cell-cell recognition	1,84E-01	3,26E+01	2	CCR7|CD6
63	GO:0001819	positive regulation of cytokine production	2,14E-01	3,73E+01	3	CCR7|CD3E|CD6
64	GO:0002429	immune response-activating cell surface receptor signaling pathway	2,17E-01	3,73E+01	3	CD3D|CD3E|FCGR2B
65	GO:0007165	signal transduction	2,58E-01	4,38E+01	6	BTLA|CCR7|CD3D|CD3E|CD6|FCGR2B
66	GO:0006954	inflammatory response	2,88E-01	4,80E+01	3	CCR7|CD6|FCGR2B
